# Effect of Viewing Disney Movies During Chemotherapy on Self-Reported Quality of Life Among Patients With Gynecologic Cancer: A Randomized Clinical Trial

**DOI:** 10.1001/jamanetworkopen.2020.4568

**Published:** 2020-05-11

**Authors:** Sophie Pils, Johannes Ott, Alexander Reinthaller, Enikoe Steiner, Stephanie Springer, Robin Ristl

**Affiliations:** 1 Department of Obstetrics and Gynecology, Medical University of Vienna, Vienna, Austria; 2 Department of Obstetrics and Gynecology, General Hospital of Vienna, Vienna, Austria; 3 Section for Medical Statistics, Center for Medical Statistics, Informatics, and Intelligent Systems, Medical University of Vienna, Vienna, Austria

## Abstract

**Importance:**

In addition to treatment efficacy, evaluation of adverse effects and quality of life assessments have become increasingly relevant in oncology.

**Objective:**

To evaluate the association of watching Disney movies during chemotherapy with emotional and social functioning and fatigue status.

**Design, Setting, and Participants:**

This randomized clinical trial was performed from December 2017 to December 2018 at a cancer referral center in Vienna, Austria. A consecutive sample of women with gynecologic cancers was recruited through July 2018. Inclusion criteria included age older than 18 years, written informed consent, and planned 6 cycles of chemotherapy with either carboplatin and paclitaxel or carboplatin and pegylated liposomal doxorubicin. Exclusion criteria were inadequate knowledge of the German language or receipt of other chemotherapy regimens. Data analysis was performed from February 2019 to April 2019.

**Intervention:**

Participants were either shown Disney movies or not during 6 cycles of chemotherapy. Before and after every cycle, they completed standardized questionnaires from the European Organisation for Research and Treatment of Cancer (EORTC).

**Main Outcomes and Measures:**

Primary outcomes were change of quality of life, as defined by the EORTC Core-30 (version 3) questionnaire, and fatigue, as defined by the EORTC Quality of Life Questionnaire Fatigue, during 6 cycles of chemotherapy.

**Results:**

Fifty-six women entered the study, and 50 completed it, including 25 women in the Disney group (mean [SD] age, 59 [12] years) and 25 women in the control group (mean [SD] age, 62 [8] years). In the course of 6 cycles of chemotherapy, patients in the Disney group felt less tense and worried less than patients in the control group according to their responses to the questions about emotional functioning (mean [SD] score, 86.9 [14.3] vs 66.3 [27.2]; maximum test *P* = .02). Furthermore, watching Disney movies was associated with less encroachment on patients’ family life and social activities, as evaluated by the social functioning questions (mean [SD] score, 86.1 [23.0] vs 63.6 [33.6]; maximum test *P* = .01). Moreover, this intervention led to fewer fatigue symptoms (mean [SD] score, 85.5 [13.6] vs 66.4 [22.5]; maximum test *P* = .01). Perceived global health status was not associated with watching Disney movies (mean [SD] score, 75.9 [17.6] vs 61.0 [25.1]; maximum test *P* = .16).

**Conclusions and Relevance:**

These findings suggest that watching Disney movies during chemotherapy may be associated with improvements in emotional functioning, social functioning, and fatigue status in patients with gynecologic cancers.

**Trial Registration:**

ClinicalTrials.gov Identifier: NCT03863912

## Key Points

**Question:**

Is watching Disney movies during chemotherapy associated with improved quality of life among patients with gynecologic cancer?

**Findings:**

In this randomized clinical trial that included 56 patients, watching Disney movies during 6 cycles of chemotherapy was associated with differences in emotional functioning, social functioning, and fatigue status scores compared with controls.

**Meaning:**

Watching Disney movies during chemotherapy may be associated with improvements in quality of life in patients with gynecologic cancer.

## Teaser

This randomized clinical trial evaluates the association of watching Disney movies during chemotherapy with emotional and social functioning and fatigue status among women with gynecologic cancers.

## Introduction

Cancer and its treatment with chemotherapy can be physically and psychologically demanding for patients. Notably, among the major worries of women with gynecologic cancer are the adverse effects of chemotherapy. Even more important, maintaining a positive attitude during treatment was a value shared by more than 90% of these patients, regardless of their age.^1^ This is in accordance with Walt Disney, who stated in 1958 that “the tonic effect of fun and play has long been recognized as an antidote to the stresses, worries, labors, and responsibilities of our workaday life.”^2^

Studies about distraction with music to reduce the stress of patients with cancer have been conducted. A Cochrane analysis^3^ found that music may have beneficial effects on quality of life (QoL), including such factors as anxiety and fatigue, in patients with cancer. Two recent studies showed impressive results: Lopez et al^4^ described that tailored music therapy intervention can help reduce global, physical, and psychosocial distress. A Danish study group^5^ demonstrated that patients with non-Hodgkin and Hodgkin lymphoma had reduced anxiety by listening to live music of their own choosing.

Furthermore, it has been shown that music can intensify emotions experienced when looking at pictures.^6^ In movies, music not only enhances the emotion of what is shown on the screen, but also can create impressions and emotions that take the audience back in time to when they heard this music for the first time and so can help recall childhood memories.^7^ Disney perfected the interaction of music and stories in his movies.^8^ In addition, studies^9,10,11^ have shown that watching Disney movies is associated with increased prosocial behavior in children and that the importance of the family is emphasized.

In patients with advanced ovarian cancer, a combination of carboplatin and paclitaxel in the adjuvant setting or carboplatin-based combination chemotherapy for recurrent disease is the standard of care.^12^ Patients with cervical or endometrial cancer benefit from platinum-based chemotherapy in combination with paclitaxel in advanced or metastatic stages.^13,14^ In addition to treatment efficacy, evaluation of adverse effects and QoL assessments have become more and more relevant.^15^ The European Organisation for Research and Treatment of Cancer (EORTC) has developed standardized questionnaires to quantify different aspects of QoL of patients with cancer, such as emotional and social functioning, as well as fatigue status.^16,17^

Disney movies provide not only the music component with their famous songs, but also provide a distraction for more than 1 hour. To the best of our knowledge, Disney movies have never been topic of an interventional study. We hypothesized that watching Disney movies might positively affect the emotional and social functioning and fatigue status and global health status of women with gynecologic cancer undergoing chemotherapy. Thus, we aimed to evaluate these issues during 6 cycles of chemotherapy in patients with gynecologic cancer who watched Disney movies during chemotherapy and controls who did not watch such movies in a pilot study.

## Methods

In this prospective randomized clinical trial, 56 patients with gynecologic cancers were recruited from December 2017 to July 2018 (the study continued until December 2018) at the Department of General Gynecology and Gynecologic Oncology, Medical University of Vienna, Vienna, Austria. The trial protocol is shown in Supplement 1.

Approval for this study was obtained from the Medical University of Vienna ethical review board. All patients provided written, informed consent. This study follows the Consolidated Standards of Reporting Trials (CONSORT) reporting guideline.

Inclusion criteria were age older than 18 years, written informed consent, and 6 planned cycles of chemotherapy with either carboplatin and paclitaxel or carboplatin and pegylated liposomal doxorubicin recommended by the tumor board according to the international guidelines.^12,13,14^ Inadequate knowledge of the German language or receipt of other chemotherapy regimens were exclusion criteria.

All eligible patients were invited to participate in the study. The patients received initial information about the study during their inpatient stay or outpatient visit before the planned chemotherapy by 1 of the physicians of the study team. On the day of the first chemotherapy, after providing signed informed consent, patients were randomized into 2 study groups: the control group included 28 patients who were not allowed to watch any show or movie during application of chemotherapy, and the Disney group included 28 patients who were shown Disney movies on portable DVD players (OK Portable DVD Player; OPD 920) during the treatment.

The aim of this study was to evaluate changes in QoL during chemotherapy.^9,10,11,18^ Therefore, emotional and social functioning, perceived fatigue, and global health status were assessed by using standardized oncologic EORTC Quality of Life Questionnaire Core-30 (QLQ-C30) (version 3) and EORTC Quality of Life Questionnaire Fatigue (QLQ-FA12) surveys. Questions concerning emotional functioning (questions 21-24), social functioning (questions 26-27), global health status (questions 29-30), and fatigue (questions 1-12) were analyzed as recommended, resulting in scores ranging from 0 to 100, with larger values indicating a better status. The EORTC QLQ-C30 Summary Score was calculated to capture additional information. In addition, data on patients’ age, cancer location, recurrence status, type of chemotherapy, and body mass index (calculated as weight in kilograms divided by height in meters squared) were collected at each therapy session. Double data entry was performed upon completion of the study.

All patients were asked to complete 2 surveys on the day of chemotherapy application. Both were given to the patients by a physician of the study team after ordering the chemotherapy. At the same time, the Disney group had to choose a movie, and the charged DVD players with disposable headphones were distributed. Patients completed the first survey while waiting for the delivery of the chemotherapy. When the nurses started the chemotherapy, patients turned on the DVD players; the nurses or the study team assisted them when needed. The second survey was taken right after chemotherapy application before patients left the oncology unit. Both surveys and, if applicable, the DVD player were returned to a box in the nurses’ station by the patients. Thus, QoL was evaluated 12 times per patient during 6 cycles of chemotherapy.

Eight movies were available (all in German language): *Cinderella* (1950), *Lady and the Tramp *(1955), *The Sword in the Stone* (1963), *Mary Poppins* (1964), *The Jungle Book* (1967), *Aristocats* (1970), *Robin Hood *(1973), and *The Little Mermaid* (1989). All the selected movies were produced between 1950 and 1989. We deliberately chose older movies because they were more likely to evoke memories of the past and have a slower storyline than newer Disney movies.^7^ Furthermore, movies with particularly sad scenes (eg, *Dumbo* and* Bambi*) were excluded. All selected stories are about strong main characters who are curious, faithful, and brave and who share in the community life with high moral values and have a happy ending.

The patients watched 6 of 8 movies entirely while the therapy was being applied. Therapy duration for carboplatin and paclitaxel was approximately 4 hours, and that for carboplatin and pegylated liposomal doxorubicin was approximately 90 minutes. The Disney movies lasted for 76 to 140 minutes (*Cinderella*, 76 minutes; *Lady and the Tramp*, 76 minutes; *The Sword in the Stone*, 79 minutes; *Mary Poppins*, 140 minutes; *The Jungle Book*, 89 minutes; *Aristocats*, 78 minutes; *Robin Hood*, 83 minutes; and *The Little Mermaid*, 85 minutes). During each cycle of chemotherapy, patients watched 1 movie. Patients chose the movies themselves. The movies started along with the beginning of chemotherapy application. To prevent distractions and disturbance, the patients were given headphones to control the volume themselves.

**Figure 1.**
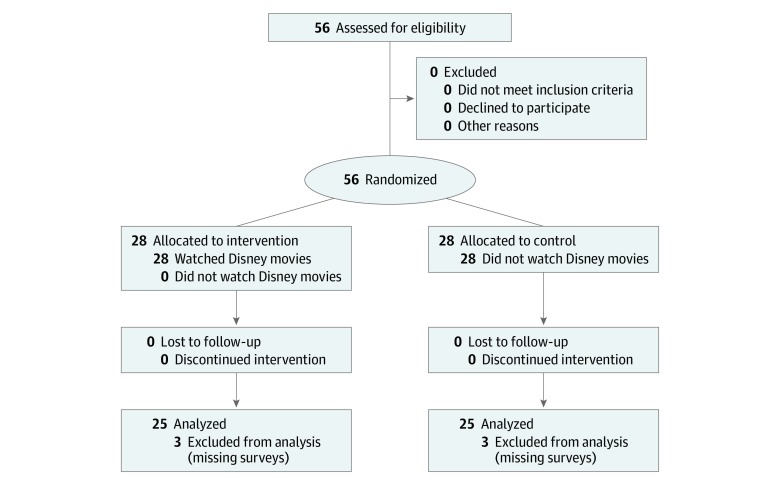
CONSORT 2010 Flow Diagram

Six women (3 of each group) had to be excluded. In the control group, 1 patient changed her appointments too often and, thus, it was impossible to hand out all the surveys to the patient in time; 1 patient completed the surveys but forgot repeatedly to return them; and 1 patient died after the third cycle of chemotherapy. In the Disney group, 1 patient did not respond to the chemotherapy, and her medication was changed after the third cycle; 1 patient found the questions in the survey too personal and quit after the first session; and 1 patient quit the study during the first movie because she was reminded of her late daughter. Those 6 patients were replaced immediately after exclusion. This resulted in a final patient population of 50 (25 patients for each group) (Figure 1). The study duration was, depending on the type of chemotherapy, up to 6 months (18 weeks for the 3-times-weekly carboplatin and paclitaxel or 24 weeks for the 4-times-weekly carboplatin and pegylated liposomal doxorubicin).

Patients and the public were not involved in the design, conduct, and reporting of the research. Sixty folders were prepared, each including a sealed envelope (containing a paper marked either *Disney* or *control*), 2 informed consent forms, 6 case report forms, and 12 numbered surveys (EORTC QLQ-C30 version 3 and EORTC QLQ-FA12). All documents were labeled in advance with the patient’s future allocation number. The folders were then deposited at both gynecologic oncology wards. The envelope was opened only after the informed consent form was signed. Research Randomizer software version 4.0 (Social Psychology Network) was used to create the primary list to assign the envelopes. To show the movies in a public noncommercial setting, licenses for 1 year were acquired from Motion Picture Licensing Company, MPLC Austria, GmbH.

### Primary Outcome Measures

#### Change of QoL During 6 Cycles of Chemotherapy 

Changes in QoL were measured before and after each chemotherapy session (at baseline and at 3, 6, 9, 12, and 15 weeks after inclusion for patients receiving carboplatin and paclitaxel or at 4, 8, 12, 16, and 20 weeks after inclusion for patients receiving carboplatin and doxorubicin). Changes in QoL during 6 cycles of chemotherapy were defined by the EORTC QLQ-C30 (version 3).

The EORTC QLQ-C30 (version 3) uses for questions 1 to 28 a 4-point scale. The scale is scored as 1 (not at all), 2 (a little), 3 (quite a bit), and 4 (very much). Half-points are not allowed. The range is 3. For the raw score, fewer points are considered to indicate a better outcome.

The EORTC QLQ-C30 (version 3) uses for questions 29 and 30 a 7-point scale. The scores range from 1 (very poor) to 7 (excellent). Half-points are not allowed. The range is 6. First, the raw score has to be calculated with mean values. Then linear transformation is performed so that the scores are comparable. More points are considered to indicate a better outcome.

#### Change of Fatigue During 6 Cycles of Chemotherapy 

Changes in fatigue were measured before and after each chemotherapy (at baseline and at 3, 6, 9, 12, and 15 weeks after inclusion for patients receiving carboplatin and paclitaxel or at 4, 8, 12, 16, and 20 weeks after inclusion for patients receiving carboplatin and doxorubicin). Change in fatigue during 6 cycles of chemotherapy was defined by the EORTC QLQ-FA12.

The EORTC QLQ-FA12 uses for questions 1 to 12 a 4-point scale. The scale is scored as 1 (not at all), 2 (a little), 3 (quite a bit), and 4 (very much). Half-points are not allowed. The range is 3. For the raw score, fewer points are considered to indicate a better outcome. The raw score has to be calculated using the mean value for questions 1 to 12.

### Statistical Analysis

Metric variables were described by mean and SD and were compared between groups using the Welch-Satterthwaite *t* test. Categorical variables were described by absolute and relative frequencies and were compared between groups using the Fisher exact test. All statistical tests were 2-sided. Longitudinally measured outcome parameters (emotional functioning, social functioning, global health status, fatigue, and the EORTC QLQ-C30 Summary Score) were analyzed in terms of regression models, explaining the expected value at visits 2 to 12 by study group, visit (regarded as categorical variable), the interaction of study group and visit, and the respective baseline value at visit 1. Robust sandwich variance estimation was applied to account for dependencies of observations within the same patient. To test the null hypothesis of equal time courses in both study groups, we performed a maximum test for the baseline-adjusted between-group differences at visits 2 to 12 as calculated from the regression model. Briefly, a standardized mean difference between the 2 groups was calculated for each visit. The maximum of these differences was used as global test statistic, and the corresponding *P* value was calculated on the basis of a multivariate t-distribution, thereby adjusting for the fact that multiple time points were assessed.^19^ The comparison using the maximum statistic was planned because this statistic is sensitive to between-group differences at any single time point and there was no a priori knowledge at which time points a potential effect would be present. The maximum tests were performed using the library mmmgee in R statistical software version 3.5.1 (R Project for Statistical Computing).^20^ All other statistical analyses were performed with SPSS statistical software version 25 (IBM).

The sample size was planned under the scenario that there would at least be a mean difference of 0.5 SD at 2 time points and a mean difference of 1 SD at 2 further time points. We further made the simplifying assumption of a moderate correlation of 0.5 between any 2 measurements of the same patient. Under this planning scenario, 22 patients per group were required to achieve 80% power at a significance level of *P* < .05 with a maximum test based on a multivariate t-distribution. The actual sample size was set at 25 per group, and we planned to recruit up to 60 patients to account for dropouts. Data analysis was performed from February 2019 to April 2019.

## Results

Baseline patient characteristics are provided in Table 1. There were no statistically significant differences between the Disney and the control group concerning age (mean [SD], 59 [12] years vs 62 [8] years; *P* = .30), body mass index (mean [SD], 24.3 [5.5] vs 26 [6.2]; *P* = .33), location of cancer (ovarian, 19 patients [76%] vs 20 patients [80%]; endometrial, 3 patients [12%] vs 4 patients [16%]; cervical or vulvar, 3 patients [12%] vs 1 patient [4%]; *P* = .77), recurrence status (yes, 9 patients [36%] vs 10 patients [40%]; *P* = .77), and type of chemotherapy (carboplatin and paclitaxel, 19 patients [76%] vs 18 patients [72%]; carboplatin and doxorubicin, 6 patients [24%] vs 7 patients [28%]; *P* > .99). In the Disney group, the mean (SD) weight difference between the initial and the last visit was 0.0 (3.7) kg, whereas patients in the control group lost a mean (SD) of 1.4 (3.9) kg (*P* = .18).

**Table 1.** Patient Characteristics

Characteristic	Patients, No. (%)		*P* value
	Disney (n = 25)	Control (n = 25)	
Age, mean (SD), y	59 (12)	62 (8)	.30
Body mass index, mean (SD)	24.3 (5.5)	26 (6.2)	.33
Cancer			
Ovarian	19 (76)	20 (80)	.77
Endometrial	3 (12)	4 (16)	
Cervical or vulvar	3 (12)	1 (4)	
Chemotherapy			
Carboplatin and paclitaxel	19 (76)	18 (72)	>.99
Carboplatin and doxorubicin	6 (24)	7 (28)	
Recurrence status, yes	9 (36)	10 (40)	.77
Weight loss during 6 cycles of chemotherapy, mean (SD), kg	0.0 (3.7)	−1.4 (3.9)	.18

Body mass index is calculated as weight in kilograms divided by height in meters squared.

The pastime used during the 6 treatments was evaluated in the control group, and multiple answers were possible. Sleeping had the highest prevalence (38%), followed by readings books or magazines (29%), playing with smartphones (10%), solving brainteasers (9%), talking to other patients or visitors (7%), listening to music (6%), and knitting (1%).

**Figure 2.**
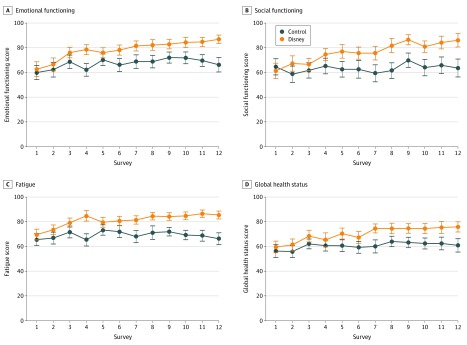
Emotional Functioning, Social Functioning, Fatigue, and Global Health Status Scores During 6 Cycles of Chemotherapy in Patients Watching Disney Movies or Not Graphs show mean scores for emotional functioning (A), social functioning (B), fatigue (C), and global health status (D). Odd-numbered visits represent prechemotherapy survey, and subsequent even-numbered visits represent postchemotherapy surveys on the same day. Error bars correspond to 1 SE of the mean. The maximum test *P* values for the between-group comparison of mean trajectories were *P* = .02 (A), *P* = .01 (B), *P* = .01 (C) and *P* = .16 (D).

After 6 cycles of chemotherapy, patients in the Disney group felt less tense, irritable, depressed, and worried compared with patients in the control group (mean [SD] emotional functioning score at the final visit, 86.9 [14.3] vs 66.3 [27.2]; maximum test *P* = .02) (Table 2 and Figure 2A). Furthermore, watching Disney movies seemed to be associated with less encroachment on patients’ family life and social activities, as evaluated by the social functioning questions (mean [SD] score, 86.1 [23.0] vs 63.6 [33.6]; maximum test *P* = .01) (Table 2 and Figure 2B). Moreover, this intervention led to fewer symptoms of fatigue, such as exhaustion, tiredness, and frustration, so patients felt less helpless (mean [SD] fatigue scores, 85.5 [13.6] vs 66.4 [22.5]; maximum test *P* = .01) (Figure 2C). Perceived global health status was not affected by watching Disney movies (mean [SD] score, 75.9 [17.6] vs 61.0 [25.1]; maximum test *P* = .16) (Table 2 and Figure 2D). Although the outcome values improved over the 6 cycles of chemotherapy, there was no difference in measurements before and after intervention on the same day (Figure 2). In addition, the EORTC QLQ-C30 Summary Score was calculated and showed a statistically significant increase in QoL, with mean (SD) scores before the first chemotherapy of 72.0 (17.1) in the Disney group vs 69.3 (19.5) in the control group and scores after the last chemotherapy of 88.4 (10.1) in the Disney group vs 70.0 (18.7) in the control group (*P* = .001).

**Table 2.** Emotional and Social Functioning and Global Health Status Scores Before the First and After the Last Chemotherapy, Comparison of Disney Group and Control Group

Variable	Score, mean (SD)		
	Disney (n = 25)	Control (n = 25)	General population norm data^a^
Before the first chemotherapy			
Emotional functioning	62.7 (30.2)	60.0 (28.1)	71.0 (24.1)
Social functioning	61.3 (31.1)	64.7 (32.7)	83.8 (25.7)
Global health status	59.7 (23.7)	56.3 (26.1)	62.6 (22.5)
After the last chemotherapy			
Emotional functioning	86.9 (14.3)	66.3 (27.2)	71.0 (24.1)
Social functioning	86.1 (23.0)	63.6 (33.6)	83.8 (25.7)
Global health status	75.9 (17.6)	61.0 (25.1)	62.6 (22.5)

^a^ General population is defined as women aged 50 to 59 years.^14^

## Discussion

When comparing the course of patients’ answers on their emotional functioning, social functioning, and fatigue status from the first treatment to the last, the participants in the Disney group felt better than those in the control group. Moreover, when comparing the results with the normative data for the EORTC QLQ-C30 in nononcologic women aged 50 to 59 years, it is remarkable that, before the intervention, both the control group and the Disney group rated their emotional and social functioning and their global health status below the levels of the published data, whereas only the Disney group rated their status above the levels of the published data after the sixth chemotherapy session and Disney movie.^21^ Thus, patients in the Disney group perceived their QoL after 6 cycles of chemotherapy higher than healthy controls in the same age range (Table 2). This is a surprising result, which we find hard to interpret. Hypothetically, it might be associated with a short-term effect of the movies on patients’ self-perception and hope. The latter has already been claimed to be positively correlated with QoL in adult oncologic patients.^22^ Notably, EORTC QLQ-30 surveys in nononcologic women watching Disney movies have never been evaluated, to our knowledge. However, the surprisingly high final QoL in the Disney group might also be associated with the small sample size.

Most studies about QoL in oncologic patients were conducted with music intervention and showed ambivalent results.^3,4,5^ Disney movies go a step further, because they provide not only the music component with the famous songs, which are generally considered quite catchy but also tell exciting stories.^8^ Furthermore, the duration of a Disney movie is 76 to 140 minutes, whereas most interventional music sessions last only 30 to 45 minutes.^3,4,5^ Thus, Disney movies distracted patients for at least one-third of the duration of the treatment. Moreover, movies affect more senses than music alone.^6,23^

In addition to providing distraction, it is interesting to note that Disney movies tell stories of surmounting difficulties without necessarily resolving them. They are more about accepting change than about heroically overcoming all odds. The stories tend to have a happy ending, but usually a bittersweet one. There is drama and sadness and, eventually, things improve. However, the true victory of the characters is their personal growth. Frequently, characters mature and become adults.^10^ This transition is often painful and difficult, but when it is resolved, things get better. Death and loss are omnipresent in Disney movies, and they are even applied to teach children to cope with tragedy.^24^ In his review of the movie *Monsters, Inc*, the author Dave Kehr writes, “Walt Disney once said that for every laugh there should be a tear, and I’ve always believed very strongly in that.”^25^

It is not known whether Walt Disney created so many motherless characters to deal with his perceived guilt for his mother’s death. However, known positive coping strategies, such as accepting support from friends and loved ones or active problem-solving, are frequently found in his movies.^11,18^ This may be a reason for the increase in social functioning scores in our data. On the other hand, coping mechanisms such as escape and distraction are seen as helpful.^26^ Because watching Disney movies might take the patients’ minds off their treatment, this could explain the increase in emotional functioning scores.

Furthermore, Viktor Frankl, a psychologist and survivor of the Shoah, described a possible strategy to cope with tragedy, whereby a person thinks back to the “granaries of the past,”^27^ to simpler, friendlier times full of hope. We have deliberately chosen older movies to bring patients into the happier past.^7^ There is a certain nostalgia about Disney movies, with their familiar characters and songs, that may help alleviate the fear of the present. Even if we can admit that our patients already have developed coping mechanisms in their past, offering new ones, to have a wider choice, could also explain the findings.^28^

Finally, Disney movies present 2 additional interesting aspects: first, Disney movies have become part of international culture and are instantly recognizable in most places and societies and, thus, have a low cultural entry level. This makes them easier to apply than other, more specific works of art.^5^ Second, the movies contain catchy songs, likable characters, and memorable phrases and scenes. This may bring back a feeling of relief.^10^ Moreover, watching movies on a portable DVD player is an easy and affordable tool.

### Limitations

The fact that only women were included in this study is a minor study limitation, because no conclusions can be drawn for male oncologic patients. Moreover, no third study group that watched other movies was included. Furthermore, because of the single center setting, only 56 patients could be included during the 7 months of the recruitment period. In addition, it should be noted that, for patients with deceased children, watching Disney movies could have a negative effect. Also, because the study ended on the day of the last chemotherapy session, no conclusions on the persistent effect of these movies can be drawn. However, all study patients were treated by the same group of physicians, limiting a potential emotional bias. In addition, the fact that the control group was not allowed to watch any movies at all might be considered restrictive and unrealistic and should be considered a minor study limitation.

## Conclusions

These findings suggest that watching Disney movies during chemotherapy is associated with improved emotional functioning, social functioning, and fatigue status in patients with gynecologic cancer. We consider the results of this study more than promising and suggest that affected patients can be counseled on the basis of the data.

## Notes

Supplement 1. **Trial Protocol**

Supplement 2. **Data Sharing Statement**
