# Supplementary material for: Effect of Viewing Disney Movies During Chemotherapy on Self-Reported Quality of Life Among Patients With Gynecologic Cancer: A Randomized Clinical Trial
Source: JAMA Netw Open. 2020 May 11;3(5):e204568. doi: 10.1001/jamanetworkopen.2020.4568 (PMC7215261; doi:10.1001/jamanetworkopen.2020.4568)
Supplement: Supplement 1. — Trial Protocol [file jamanetwopen-3-e204568-s001.pdf]

Study protocol

Prospektive Studie über den Einfluss von Disney Filmen während Chemotherapie auf die Lebensqualität von gynäkoonkologischen Patientinnen

Impact of Disney movies during chemotherapy on QOL of gyneco-oncologic patients, a prospective study

Version: 1.2 from 06.11.17

Submitter: Dr. Sophie Pils

Assoc. Prof. Dr. Johannes Ott

Univ. clinic for gynaecology, MUW

Correspondence:

Dr. Sophie Pils

Department of Gynecology and Obstetrics

Medical University of Vienna

A-1090 Vienna

Phone: 01-40400-29150

Fax: 01-40400-29110

E-mail: sophie.pils@meduniwien.ac.at

Summary

Evaluation of the influence of Disney films on the quality of life of oncological patients.

Background

The focus of oncological therapies is primarily on efficacy and secondarily on the side effect profile. In recent years, more and more studies on the quality of life (QOL) of oncological patients have been conducted.

There are studies that measure QOL under standard therapy (1,2) as well as studies that compare QOL under new drugs with standard therapy (3). Occasionally, alternative therapies were evaluated in addition to known therapies (4). The majority of these studies use the validated EORTC C30 (European Organization for Research and Treatment of Cancer-Gynecologic Cancer Group) (5) Quality of life questionnaire to evaluate the quality of life of the patients.

Oncological patients in the gynecological oncology department are admitted to the planned chemotherapy ward either in a day clinic or in an in-patient setting. During the morning doctor's visit, patients are proactively asked about side effects and the ECOG Scale of Performance Status (6) is determined. Subsequently, the appropriate chemotherapies are ordered with adequate laboratory values. The patients then wait one to two hours for the therapies. Depending on the schedule, chemotherapy is administered over a period of 3 to 5 hours. After the application of the therapies, the patients, after checking the vital parameters, are discharged into home care the same or next day, depending on the chemotherapy regimen.

Supportive measures in pediatric oncology such as "Cliniclows" and similar are socially accepted, but hardly evaluated. Patch Adams also owes us study results (7).

Disney films have been evaluated in some small studies on prosocial behavior (8). The influence of Disney films on QOL has not yet been investigated, but it can be assumed that a positive mood remains.

Disney films are translated into almost every language and thus studies are internationally comparable with this material.

Hypothesis

Disney films have a positive effect on the mood of gynecological patients and improve the subjective quality of life.

Primary aim of the study:

to evaluate whether patients who watch Disney films during chemotherapy show a better QOL after therapy than before.

Secondary study objective:

to compare the routinely evaluated allergic reactions and side effects during chemotherapy between the Disney Film Group and the control group

Methods

All patients admitted to the ward of the gynecological oncology 16E or 16D for the planned first cycle of chemotherapy (carboplatin/ paclitaxel or carboplatin/pegylated liposomal doxorubicin) will be offered study participation.

60 patients can be enrolled until 50 patients have completed the study.

Patients are randomized between the two study groups: watching Disney movies or not during the planed six cycles of chemotherapy.

Patients in the control group are asked not to watch television or use films on other technical aids. All other activities (listening to music, reading, playing cards, games of any kind, conversations with confidants...) are documented, but are not grounds for exclusion.

Inclusion criteria:

- Planned chemotherapy with 6 cycles of carboplatin and paclitaxel or carboplatin and pegylated liposomal doxorubicin at the gynecologic wards 16E and 16D of the Department for Gynecology and gynecologic oncology for Gynecology
- Age over 18 years
- Sufficient knowledge of German

Exclusion criteria:

- All other chemotherapy protocols
- Age under 18 years
- Insufficient knowledge of German

After detailed patient education and written informed consent, the patients are included in the study.

Patients are asked to complete the same questionnaires twice on the same day:

- once after ordering but before the application of chemotherapy, and
- a second time after the application of chemotherapy during which, depending on the randomization, a Disney film was shown or the patient was not allowed to watch television.

This is repeated in each of the 6 planned chemotherapy cycles.

Duration of study participation: 6 cycles of chemotherapy (depending on laboratory and side effects 6x3=18 weeks up to 6 months)

In addition, the routinely collected side effect profiles and the ECOG status are documented.

Both The Walt Disney Company, Germany, Switzerland & Austria and Motion Picture Licensing Company, MPLC Austria GmbH were informed about this planned study.

A license, which includes a non-commercial license to screen Disney films, will be acquired by Dr. Sophie Pils in case of a positive ethics vote at the beginning of the study.

The following films will be shown as part of this study: Lady and the Tramp, The Sword in the Stone, Mary Poppins, The Jungle Book, Aristocats, Robin Hood, Cinderella and The Little Mermaid.

Questionnaire

The validated EORTC QLQ-C30 (Appendix 1) and the EORTC QLQ-FA12 (Appendix 2) are used to evaluate QOL and fatigue. In addition, patients are asked to complete the EORTC QLQ - OV28 (Appendix 3), EORTC QLQ-CX24 (Appendix 4) or EORTC QLQ-EN 24 (Appendix 5) corresponding to the underlying disease.

Answering the questionnaires takes approximately 30 minutes.

If any ambiguities arise during the answering of the questionnaire, the patients will be offered help by Dr. Sophie Pils and the medical team of the gynecological department are available.

Risk-benefit evaluation

Oncological patients are routinely admitted to hospital every 3 to 4 weeks for 1 to 2 days. During these stays there are longer waiting times for the patients: waiting for the visit, waiting for the chemotherapy, sitting still or lying down while the chemotherapy is administered. In the days following chemotherapy there is fatigue, nausea, blood count disorders, neuropathies.

The aim of this study is to interrupt the patients' routine and to evaluate whether this has an influence on their quality of life.

It can be assumed that Disney films will have no negative effect on the quality of life of the patients.

The subjective quality of life of gynecological patients under chemotherapy is particularly relevant in view of the mostly poor prognosis.

Data protection

The collected data is - in the context of anonymization - coded and entered into a Microsoft Excel® table.

Only the study team has access to the non-anonymous data.

Statistics

For data processing, the patient's initials, date of birth, underlying disease, study group, chemotherapy cycle, allergic reactions, body weight and the prestigious Disney film are provided.

The questionnaires are then digitized using Excel (Microsoft, Redmond, Washington, USA) and SPSS (Predictive Analytics Software, SPSS Inc., Chicago, IL, USA). The results (1-7) are then evaluated according to EORTC guidelines.

Metric variables are described by mean and standard deviation and compared between groups using the Welch-Satterthwaite t-test. Categorical variables are described by absolute and relative frequencies and compared between groups using Fisher’s exact test.

Longitudinal outcomes are analyzed with a regression model and a maximum test. These analyses will be made with “R”. All other statistical analyses were performed with SPSS software version 25.

A p-value of <0.05 is assumed to be significant.

References

1. Fukushi K, Narita T, Hatakeyama S, Yamamoto H, Soma O, Matsumoto T, Tobisawa Y, Yoneyama T, Imai A, Yoneyama T, Hashimoto Y, Koie T, Ohyama C. Quality-of-life evaluation during platinum-based neoadjuvant chemotherapies for urothelial carcinoma. Int J Clin Oncol. 2017 Apr;22(2):366-372.

2. Brotto L, Brundage M, Hoskins P, Vergote I, Cervantes A, Casado HA, Poveda A, Eisenhauer E, Tu D; Gynecologic Cancer Intergroup Study of NCIC Clinical Trials Group (NCIC CTG); European Organization for Research and Treatment of Cancer - Gynecologic Cancer Group (EORTC-GCG); Grupo de Investigación de Cáncer de Ovario (GEICO). Randomized study of sequential cisplatin-topotecan/carboplatin-paclitaxel versus carboplatin-paclitaxel: effects on quality of life. Support Care Cancer. 2016 Mar;24(3):1241-9.

3. Stark DP, Cook A, Brown JM, Brundage MD, Embleton AC, Kaplan RS, Raja FA, Swart AMW, Velikova G, Qian W, Ledermann JA. Quality of life with cediranib in relapsed ovarian cancer: The ICON6 phase 3 randomized clinical trial. Cancer. 2017 Jul 15;123(14):2752-2761.

4. Rithirangsriroj K, Manchana T, Akkayagorn L. Efficacy of acupuncture in prevention of delayed chemotherapy induced nausea and vomiting in gynecologic cancer patients. Gynecol Oncol. 2015 Jan;136(1):82-6.

5. European Organization for Research and Treatment of Cancer–Gynecologic Cancer Group http://groups.eortc.be/qol/eortc-qlq-c30

6. ECOG Scale of Performance Status. Eastern Cooperative Oncology Group. http://ecog-acrin.org/resources/ecog-performance-status

7. Adams P. Humour and love: the origination of clown therapy. Postgrad Med J. 2002 Aug;78(922):447-8

8. Padilla-Walker LM, Coyne SM, Fraser AM, Stockdale LA. Is Disney the Nicest Place on Earth? A Content Analysis of Prosocial Behavior in Animated Disney Films. Journal of communication 63(2013)393-412.
