# Supplementary material for: Effect of Viewing Disney Movies During Chemotherapy on Self-Reported Quality of Life Among Patients With Gynecologic Cancer: A Randomized Clinical Trial
Source: JAMA Netw Open. 2020 May 11;3(5):e204568. doi: 10.1001/jamanetworkopen.2020.4568 (PMC7215261; doi:10.1001/jamanetworkopen.2020.4568)
Supplement: Supplement 2. — Data Sharing Statement [file jamanetwopen-3-e204568-s002.pdf]

Data Sharing Statement

Pils. Effect of Viewing Disney Movies During Chemotherapy on Self-Reported Quality of Life Among Patients With Gynecologic Cancer. *JAMA Netw Open*. Published May 11, 2020.
10.1001/jamanetworkopen.2020.4568

Data
Data available: Yes
Data types: Deidentified participant data, Data dictionary
How to access data: Proposals should be directed to the corresponding author, johannes.ott@meduniwien.ac.at.
When available: With publication

Supporting Documents
Document types: Informed consent form
How to access documents: Proposals should be directed to the corresponding author, Johannes.ott@meduniwien.ac.at.
When available: With publication

Additional Information
Who can access the data: Data will be available immediately after publication until 10 years.
Types of analyses: for any purpose
Mechanisms of data availability: with a signed data access agreement
Any additional restrictions: none
